# Terahertz Radiation Driven Nonlinear Transport Phenomena in Two-Dimensional Tellurene

**DOI:** 10.1021/acs.nanolett.4c05279

**Published:** 2024-12-25

**Authors:** E. Mönch, M. D. Moldavskaya, L. E. Golub, V. V. Bel’kov, J. Wunderlich, D. Weiss, J. V. Gumenjuk-Sichevska, Chang Niu, Peide D. Ye, S. D. Ganichev

**Affiliations:** † Physics Department, University of Regensburg, 93040 Regensburg, Germany; ‡ Institute of Physics, Czech Academy of Sciences, Cukrovarnická 10, 162 00 Praha 6, Czech Republic; ¶ Johannes Gutenberg-University Mainz, D-55128 Mainz, Germany; § V. Lashkaryov Institute of Semiconductor Physics, National Academy of Science, 03028, Kyiv, Ukraine; ∥ Elmore Family School of Electrical and Computer Engineering, Purdue University, West Lafayette, Indiana 47907, United States; ⊥ Birck Nanotechnology Center, Purdue University, West Lafayette, Indiana 47907, United States; # CENTERA Laboratories, Institute of High Pressure Physics, PAS, 01-142 Warsaw, Poland

**Keywords:** two-dimensional tellurene, terahertz radiation, photocurrents, photogalvanic effect, nonlinear Hall effect, Berry curvature dipole

## Abstract

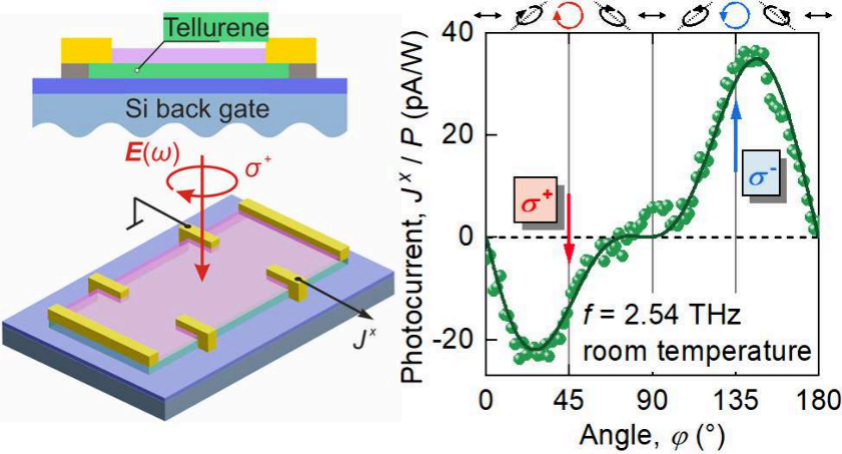

Nonlinear electron transport induced by polarized terahertz radiation is studied in two-dimensional tellurene at room temperature. A direct current, quadratic in the radiation’s electric field, is observed. Contributions sensitive to radiation helicity and polarization orientation as well as polarization independent current are found. We show that these contributions can be modified by the magnitude of the external gate potential. We demonstrate that this terahertz-driven electric current arises from the Berry curvature dipole and the side-jump microscopic mechanisms.

Tellurene, the two-dimensional (2D) manifestation of tellurium, has been recently synthesized, adding a fascinating new member to the family of elemental van der Waals materials. Its discovery marks a significant step forward in expanding the capabilities and applications of 2D materials.^1−5^ Tellurene exhibits a range of exceptional properties, including remarkable stability and catalytic activity,^6^ tunable carrier density and type via electric gating,^1^ strain-adjustable bandgap,^7^ efficient piezoelectric behavior,^8,9^ anisotropic photoresponse,^10^ low thermal conductivity, and high carrier mobility—reaching about thousand cm^2^/(V s) at room temperature.^11^ With its outstanding properties, tellurene is destined for use in cutting-edge devices such as photodetectors,^12^ modulators,^2^ saturable absorbers, mode-locking lasers,^13^ and field-effect transistors.^11^ Beyond its practical applications, tellurene has also demonstrated fundamental phenomena, including the quantum Hall effect,^14,15^ spin Hall effect,^16^ weak antilocalization,^17^ and nonlinear magnetoresistance.^18,19^ While linear transport phenomena in tellurene remain the primary focus of current research, nonlinear effects—proven to be powerful tools for probing nonequilibrium electronic processes and revealing fundamental properties—are far less explored. Recently, a significant breakthrough was achieved with the detection of a giant nonlinear Hall effect (NLH) in tellurene, highlighting its remarkable nonlinear response.^20,21^ The direct current (dc) generated in response to a static electric field ***E*** is expressed by the following relation:
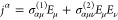
1Here  and  are the first (linear conductivity) and second (nonlinear conductivity) order in electric field dc conductivity tensors, respectively, and α, μ, and ν run over in-plane Cartesian coordinates. This second-order effect comprises both the transverse NLH *j*^*y*^ = σ_*yxx*_^(2)^*E*_*x*_^2^ and, the less-studied nonlinear longitudinal current (NLL) *j*^*x*^ = σ_*xxx*_^(2)^*E*_*x*_^2^.

The NLH was first proposed in ref. (^22^), where it was shown that in metals lacking inversion symmetry a Hall-like current due to the Berry curvature dipole in momentum space can emerge. This effect has since been extended to other noncentrosymmetric materials, including semiconductors, attracting increasing attention.^23−35^ Investigating these phenomena at high frequencies, comparable to the momentum relaxation rate, promises to reveal new effects and offer a powerful tool for studying nonlinear transport.

In this letter, we present the observation and investigation of nonlinear electron transport phenomena in tellurene, driven by terahertz (THz) radiation at room temperature. In our high-frequency experiments, the resulting dc current arises as a second-order response to the ac electric field ***E***_ω_(*t*) = ***E*** exp(−*iωt*) + E* exp(*iωt*), where ω is the driving frequency. This current is given by
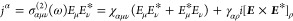
2where **χ̂**(ω) and **γ̂**(ω) are the third and second rank tensors, respectively. While the first term is a high-frequency counterpart of the second one in eq 1, the dc current described by the second one has an opposite sign for clockwise and counterclockwise rotating fields and not detectable in standard dc transport experiments. The latter one has been previously studied in bulk tellurium for both direct optical intersubband as well as indirect intraband transitions^36−38^ and is called the circular photogalvanic effect (CPGE).^39−41^ The first term on the right-hand side of eq 2 represents the linear photogalvanic effect (LPGE),^39−41^ a dc current generated by linearly polarized radiation that is sensitive to the orientation of the in-plane THz electric field. As detailed below, we observe both types of currents in tellurene under THz illumination. We attribute the THz-induced electric currents to two key microscopic mechanisms, arising from the low spatial symmetry of tellurene: the intrinsic contribution from the Berry curvature dipole and an extrinsic contribution caused by electron wave packet side-jumps during momentum scattering.

Our 2D Te flakes were synthesized by the hydrothermal growth method,^14^ for details see also Supporting Information (SI). Figure 1 shows the typical cross-section of a Te device [panel (a)] and the top view of sample #A [panel (b)]. The micrograph of the second sample under investigation (sample #B) is shown in Figure S1 in SI. The Hall bar is oriented along the *c*-axis of the tellurium, while the *a*-axis is perpendicular to it. In the following, they correspond to the *y*- and *x*-directions, respectively.

**Figure 1:**
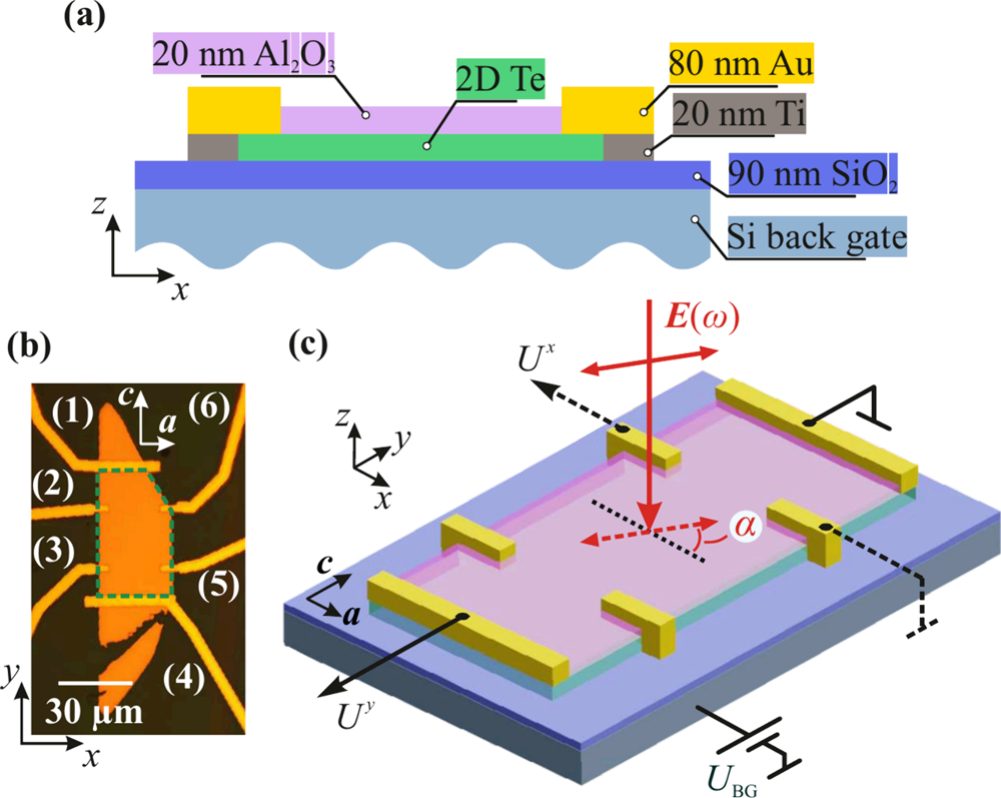
Panel (a) shows a typical cross-section of the 2D Te devices under investigation. Panel (b) shows an optical micrograph of sample #A and the corresponding contact numbering. The green dashed pentagon highlights the Hall bar. Panel (c) shows a schematic of the photovoltage measurement shown as an example for linearly polarized radiation. The dashed line marks the initial polarization state, i.e., α = 0 and φ = 0. To change the concentration and carrier type, a voltage *U*_BG_ is applied to the back gate.

The tellurene samples were investigated at room temperature (*T* ≈ 300 K). First the samples were characterized by standard electron transport as a function of the applied back gate voltage. The four-terminal resistance lies in the range of 80 to 100 kΩ at the charge neutrality point (CNP), while the two-point resistance is about twice as high. For the investigated devices, the CNP was found at high negative gate voltages (*U*_BG_ ≈ −10 V). In the following considerations, the applied back gate voltage is presented as an effective voltage with *U*_BG,eff_ = *U*_BG_ – *U*_CNP_, where *U*_CNP_ is the voltage at which the CNP appears. Consequently, for positive and negative *U*_BG,eff_ we have electron and hole type conductivity, respectively.^7^ The carrier concentration at room temperature and an effective gate voltage of 20 V is about 1 × 10^13^ cm^–2^, and the mobility μ is about 600 cm^2^/(V s) for both electrons and holes.

To excite photocurrents, monochromatic radiation with a frequency *f* = 2.54 THz (λ = 118 μm and *E*_ph_ = *hf* = 10.5 meV) was generated by an optically pumped continuous wave (*cw*) molecular gas laser. The radiation was focused by off-axis parabolic mirrors and applied to the sample under normal incidence to the sample while controlling the polarization state, i.e., the direction and ellipticity of the electric field vector ***E***_ω_, with λ/2 or λ/4 plates. The initial state of polarization (α = 0, φ = 0) points across the Hall bar, i.e., along the *x*-axis, see the black dashed line in Figure 1(c). The laser beam profile at the sample position, measured with a pyroelectric camera ,^42,43^ had a Gaussian shape with a full width at half-maximum of *d* = 0.9 mm and reaches intensities up to *I* ≈ 6.5 W cm^–2^. The area of the laser spot was much larger than the areas of the flakes, confirming uniform irradiation.

The induced photovoltage drop was measured along the *a* and *c*-axes obtained via the contact pairs 2–6 (*U*^*x*^) and 1–4 (*U*^*y*^), respectively, see Figure 1(b). In all experiments the voltage drop was generated without applying bias to the sample. The signals were measured using standard lock-in techniques locked to the chopper frequency, which modulates the *cw* THz radiation with *f*_chop_ = 130 Hz. The corresponding photocurrents *J* are related to the photovoltages *U* according to *J* = *U*/*R*_S_ where *R*_S_(*U*_BG,eff_) is the sample resistance in the 2-6 or 1-4 directions, for *U*^*x*^ and *U*^*y*^, respectively.

Photocurrents induced by circularly polarized radiation were observed over the entire gate voltage range investigated in this work. Figure 2 shows the dependence of the photocurrent in samples #A and #B measured along (*J*^*y*^) and perpendicular (*J*^*x*^) to the Hall bar. The photocurrent in response to left- and right-handed circularly polarized radiation has different magnitudes, and for some back gate voltages even changes its direction, see, e.g., Figure 2(a), (e), and (f). These dependencies are obtained by rotating a lambda quarter plate, which changes the degrees of linear and circular polarization, see polarization ellipses above Figure 3. We have fitted the dependencies as a sum of the Stokes parameters^44^ describing the polarization states of the radiation, using different weights as fitting parameters, see discussion below and SI for details:
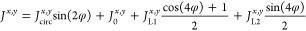
3where *J*_circ_^*x*,*y*^ is the amplitude of the circular photocurrent, *J*_0_^*x*,*y*^ is that of the polarization independent contribution, and *J*_L1_^*x*,*y*^, *J*_L2_^*x*,*y*^ are the amplitudes of the photocurrent in response to the linearly polarized radiation. Note that for right- (σ^+^, φ = 45°) and left-handed (σ^–^, φ = 135°) polarization the last two terms in eq 3 vanish and only circular and polarization independent photocurrents contribute to the signal.

**Figure 2:**
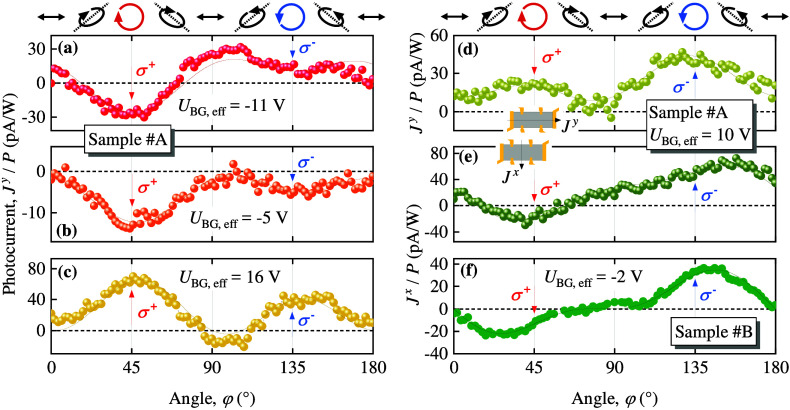
Dependencies of the photocurrent (colored circles) on the rotation angle of the λ/4 wave plate. The data are shown for several examples of effective back gate voltages ranging from hole (*U*_BG,eff_ < 0) to electron conductivity (*U*_BG,eff_ > 0). The photoresponse is normalized to the laser power incident on the sample. Panels (a)-(c) show normalized photocurrent, *J*^*y*^/*P*, obtained for sample #A. Panels (d) and (e) show *J*^*y*^/*P* and *J*^*x*^/*P* measured for *U*_BG,eff_ = 10 V measured in sample #A. Panel (f) shows the data for sample #B. The red and blue arrows in each panel mark the right-handed (σ^+^) and left-handed (σ^–^) circular polarization states, respectively. At the top, the polarization ellipses corresponding to some angles φ are shown. The solid lines represent the corresponding fits according to eq 3. The fit coefficients are listed in Tab. S1 for sample #A and in Tab. S2 for sample #B in SI. The inset illustrates the measurement direction of the photocurrent, *J*^*x*^ and *J*^*y*^, with respect to the Hall bar geometry.

**Figure 3:**
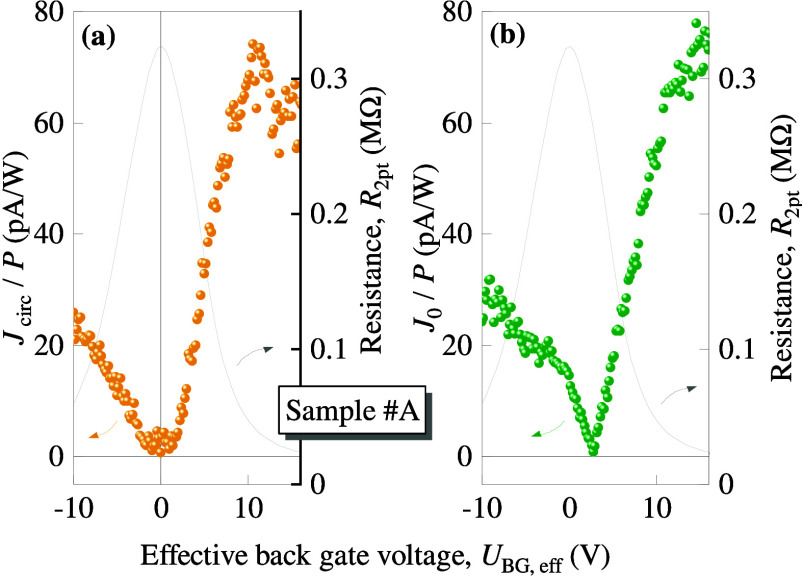
Back gate voltage dependencies of the circular *J*_circ_, panel (a), and polarization independent *J*_0_, panel (b), photocurrent contributions calculated from the measured projections of the individual photocurrents obtained in *x*- and *y*-directions as  and . The gray trace represents the corresponding two-point resistance (right axes).

As we discuss below, all photocurrent contributions are caused by nonlinear transport phenomena and defined by the symmetry of the system. The latter is the lowest for tellurene, which has no nontrivial symmetry elements. For this reason, the direction of the induced photocurrent is arbitrary. Therefore, for the analysis of its functional behavior, in our case the dependencies on the back gate voltage, it is convenient to plot the amplitudes of the photocurrents calculated from their projections in the two perpendicular directions along *x* and *y*. For example, the magnitude for the circular photocurrent is defined as . The gate voltage dependencies of the circular and the polarization independent photocurrent are shown in Figure 3 together with the sample resistance. Both photocurrent contributions behave similarly: starting from the hole conductivity region (*U*_BG,eff_ < 0) the photocurrent magnitude decreases, approaches zero and increases significantly in the region of electrons (*U*_BG,eff_ > 0). The gate voltage dependencies of the photocurrent projections are illustrated in Figure S4 in SI.

While the photocurrent induced by linearly polarized radiation contributes significantly to the polarization dependencies, obtained by rotating the λ/4 wave plate, they can be easily studied using linearly polarized radiation. Corresponding dependencies are shown exemplary in *x*-direction in Figure 4. In this experimental setup the polarization dependencies are defined as
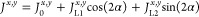
4with the same amplitudes *J*_0_^*x*,*y*^, *J*_L1_^*x*,*y*^, and *J*_L2_^*x*,*y*^ as in eq 3. Note that the conversion of eq 3 into eq 4 and vice versa is related to that of the Stokes parameters, see SI for details and, e.g., refs. (^44^) and (^45^). Figure 4 shows that increasing the back gate voltage changes both the amplitude and the phase of the polarization-dependent photocurrent. This is due to the different gate voltage dependencies of *J*_L1_ and *J*_L2_. The same behavior was observed along the *y*-direction, see the Figure S5 in SI. Similar to the photocurrents *J*_circ_ and *J*_0_ we calculate the individual gate voltage dependencies of *J*_L1_ and *J*_L2_ according to . Figure 5 shows that both contributions indeed depend differently on the gate voltage and that the photocurrent *J*_L2_ is dominating over the whole range. Note that both photocurrents approach minima near the CNP.

**Figure 4:**
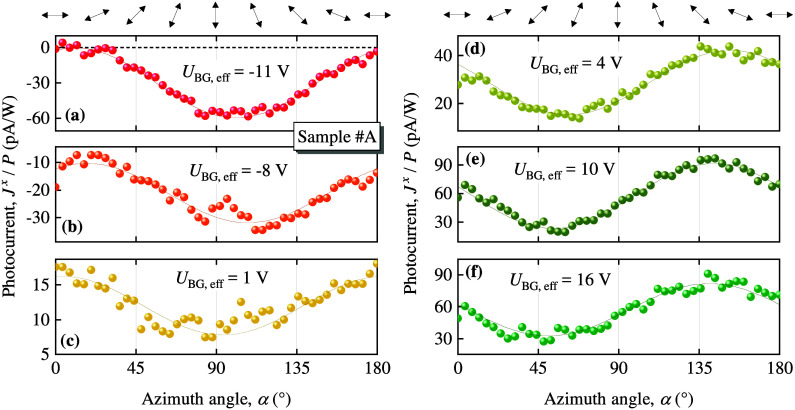
Dependencies of the photocurrent *J*^*x*^ (colored circles) on the azimuthal angle α, which defines the orientation of the electric field vector ***E*** with respect to the *x*-direction. The data are shown for several values of the effective back gate voltages ranging from hole (*U*_BG,eff_ < 0) to electron conductivity (*U*_BG,eff_ > 0). The orientations of ***E*** of the linearly polarized radiation for several angles α are shown at the top of the figure. The solid lines represent the corresponding fits according to eq 4. The fit coefficients are given in Tab. S1 in SI.

**Figure 5:**
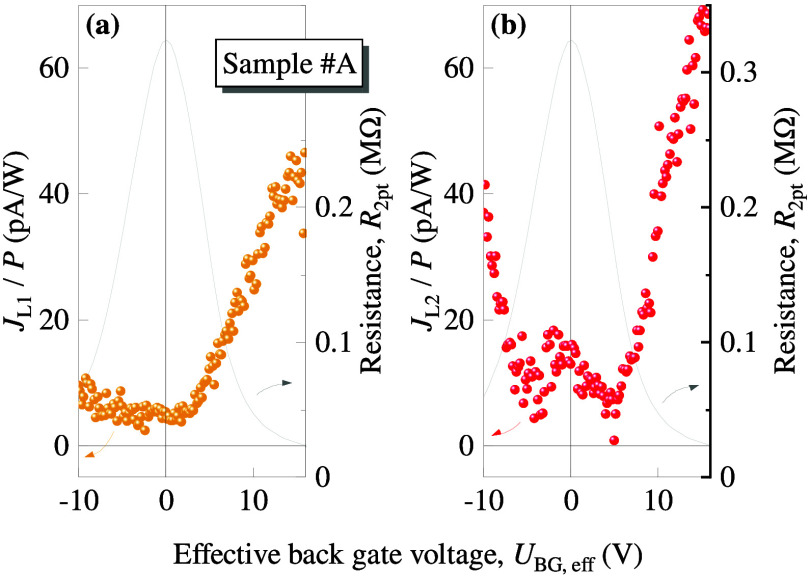
Back gate voltage dependencies of the linear photocurrent contributions, *J*_L1_ [panel (a)] and *J*_L2_ [panel (b)]. Their magnitude was calculated from the measured projections of the individual photocurrents obtained in the *x*- and *y* – directions using . The gray traces represent the corresponding two-point resistance (right axes).

Finally we emphasize that over the entire range of the available laser power, all photocurrent contributions scale linearly with the increase in radiation power *P* ∝ *E*_0_^2^, where *E*_0_ is the electric field amplitude of the radiation, see Figure S3 in SI.

In bulk Te, the linear and circular currents are expected only in certain crystallographic directions. In  2D tellurene, the point symmetry group is *C*_2_, with the only nontrivial symmetry element being rotation by the angle π about the in-plane axis  (*a* axis). The presence of a substrate makes the ±*z*-directions inequivalent, and the *C*_2_ operation does not transform the tellurene to itself. As a result, the point symmetry is reduced to *C*_1_ where any nontrivial symmetry elements are excluded. In systems with such a low symmetry excitation with homogeneous radiation at normal incidence results in a dc current, which is described by
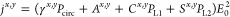
5Here *j*^*x*,*y*^ ∝ *J*^*x*,*y*^ is the photocurrent density projection on the axis *x* or *y* in the structure plane, the radiation’s electric field is ***E***_ω_ = ***e****E*_0_ exp(−*iωt*) + c.c. where ***e*** is the polarization unit vector and *E*_0_ is the amplitude, *P*_circ_ = −2Im(*e*_*x*_*e*_y_^*^), *P*_L1_ = |*e*_*x*_|^2^ – |*e*_*y*_|^2^, *P*_L2_ = 2Re(*e*_*x*_*e*_y_^*^) are the Stokes parameters of the radiation,^44^ and γ^*x*,*y*^, *A*^*x*,*y*^, *C*^*x*,*y*^, and *S*^*x*,*y*^ are eight coefficients that are linearly independent due to the absence of any nontrivial symmetry operation. Indeed, our experimental results show that all eight coefficients have comparable magnitudes, resulting in photocurrents in *a*- and *c*-directions, see Tabs. S1 and S2 in SI. Therefore, the experiments clearly demonstrate that we are dealing with a 2D system, which agrees with previous results on quantum Hall effect measurements on similar structures.^14,15^

Now we discuss the observed polarization dependencies. The introduced parameters *P*_circ_, *P*_L1_, and *P*_L2_ describe the polarization state, which varies by the rotation of the wave plates in our experiments. These parameters are given by the trigonometric functions in eqs 3 and 4 describing the polarization dependencies of the photocurrent contributions associated with *J*_circ_^*x*,*y*^, *J*_L1_^*x*,*y*^ and *J*_L2_^*x*,*y*^.^45^ Consequently, the photocurrent described by the phenomenological eq 5 is in full agreement with the experimental results showing the above polarization dependencies presented in Figures 2 and 4, as well as in Figures S5, S6, and S7 in SI. Figure 4 shows that the gate voltage changes the relation between these amplitudes: While at low negative *U*_BG,eff_ we have |*J*_L1_^*x*^| > |*J*_L2_^*x*^|, application of a high positive voltage results in the opposite relationship. This leads to a phase shift of the photocurrent dependence. This is in line with eq 5 showing that *J*_L1_^*x*,*y*^ and *J*_L2_^*x*,*y*^ are linearly independent and, thus, can be varied independently by any external parameter. Particularly interesting is the analysis of the polarization dependencies shown in Figure 4, because it allows one to clearly separate the high-frequency NLH and NLL contributions. Indeed, *J*^*x*^ measured at α = 90° (***E***∥*y*) gives the NLH current perpendicular to the radiation’s electric field, while *J*^*x*^ measured at α = 0 (***E***∥*x*) corresponds to the NLL. Figure 4 shows that they are comparable, which is not surprising, see also Tabs. S1, S2 and Figures S5, S7 in SI. Note that for *J*^*y*^, NLH and NLL are obtained for α = 0 and α = 90°, respectively.

The investigated photocurrents are excited by THz radiation with a photon energy of a few meV, which is lower than the energy gap of tellurene. Consequently, they are caused by the free carrier Drude-like radiation absorption. At room temperature in tellurene the THz radiation excites only one carrier type, either holes or electrons, depending on the gate voltage.^14^ Under these conditions, the THz-induced photocurrents given by eq 5 are described by the Boltzmann kinetic equation for the electron distribution function *f*_***k***_. Here, ***k*** is the two-dimensional electron wavevector and the equation has the following form
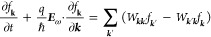
6where *W*_***k***′***k***_ is the probability of scattering between the electron states with wavevectors ***k*** and ***k***′ and *q* is the carrier charge being ± |*e*| for holes and electrons, respectively. The electric current density is calculated as
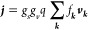
7where *g*_*s*_ and *g*_*v*_ are the spin and valley degeneracies, and ***v***_***k***_ is the electron velocity. The distribution function *f*_***k***_ is quadratic in *q****E***_ω_, corresponding to the photocurrent’s linear dependence on the radiation power observed in the experiment. Equation 7 also shows that the photocurrent is proportional to the third power of the carrier charge. Consequently, it changes its direction when passing through the CNP and approaches zero value at *U*_BG,eff_ = 0. The latter is evident from the experimental data, see Figures 3 and S4 in SI.

In the semiclassical approach, there are three microscopic mechanisms of THz radiation-induced photocurrents. As mentioned in the introduction, these are the intrinsic Berry curvature dipole (BCD) mechanism and extrinsic contributions due to side jumps of electron wave packets occurring in momentum scattering and skew scattering processes.^27,46^ The BCD mechanism of the photocurrent is accounted for in the anomalous velocity linear in the electric field of the radiation
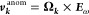
8Here, the Berry curvature is **Ω**_***k***_ = **∇**_***k***_ × *i*⟨*u*_***k***_|∇_***k***_*u*_***k***_⟩, where *u*_***k***_ is the electron Bloch amplitude for the energy band in which the radiation is absorbed. Finding the correction to the distribution function *f*_1_ ∝ *E*_ω_ from eq 6 with an ordinary scattering probability , the BCD contribution to the photocurrent is calculated via eq 7 with ***v***_***k***_ = ***v***_***k***_^anom^. For elastic scattering, , where ε_***k***_ is the energy dispersion in the band, and  is the disorder potential correlator. The considered mechanism is responsible for all detected currents: circular, linear, and polarization independent. Note that, as can be seen from eq 8, the BCD mechanism at linear polarization contributes only to the perpendicular electric current, i.e., to the NLH. We also find that the circular photocurrent is present at frequency ω comparable or higher than the transport relaxation rate.

Another photocurrent mechanism is the side jump caused by the electron wavepacket shifts ***r***_***k***′***k***_ that occur during scattering and is given by
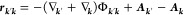
9where Φ_***k***′***k***_ is the phase of the matrix element of the scattering ***k*** → ***k***′, and the Berry connection ***A***_***k***_ = *i*⟨*u*_***k***_|∇_***k***_*u*_***k***_⟩.

There are two contributions to the photocurrent due to side jumps. One comes from the side-jump accumulation, which leads to the velocity correction
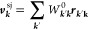
10Then by finding the correction to the distribution function *f*_2_ ∝ *E*_ω_^2^ from the Boltzmann eq 6 with the scattering probability , the side-jump accumulation contribution to the photocurrent is calculated by eq 7 with ***v***_***k***_ = ***v***_***k***_^sj^. This contribution is insensitive to the radiation helicity and contributes to the constants *A*^*x*,*y*^, *C*^*x*,*y*^ and *S*^*x*,*y*^ in eq 5, i.e., to the polarization-independent and linear polarization dependent photocurrents.

Another correction to the distribution function comes from the linear in the electric field scattering probability  caused by the correction to the energy conservation law due to the work of the field at the side jump:
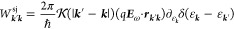
11Iterating the kinetic eq 6 with  in *E*_ω_, one finds the so-called anomalous distribution *f*^adist^ ∝ *E*_ω_^2^. Then the corresponding contribution to the photocurrent is calculated by eq 7 with *f*_***k***_ = *f*^adist^ and an ordinary velocity ***v***_***k***_ = *ℏ*^–1^***∇***_***k***_ε_***k***_. This mechanism also gives rise to all observed photocurrent contributions including the helicity dependent photocurrent.

Last but not least, the linear photocurrent can also be caused by skew scattering, previously considered for dc NLH and LPGE.^24,47^ In the case of the circular photocurrent, BCD and side-jump contributions dominate for THz frequencies and room temperature, see SI.

Finally, we briefly discuss the observed gate voltage dependencies for the circular and polarization independent photocurrents, see Figure 3. While the dependencies are similar, the positions of the photocurrent nodes are different. We attribute this to the fact that *J*_circ_ and *J*_0_ are caused by fundamentally different microscopic mechanisms as analyzed above. A detailed consideration of their interplay is given in SI.

To sum up, the obtained expressions describe nonlinear transport in 2D tellurene. As mentioned above, they yield circular and polarization-independent photocurrents (see Figures 2, 3, S4, S6, and S8 in SI), as well as linear photocurrents (see Figures 4, 5, S5, and S7 in SI). [Note that the circular photocurrent (CPGE) excited by infrared radiation with photon energy exceeding the energy gap and caused by direct interband optical transitions has been observed in ref. (^48^).] While the BCD mechanism at linear polarization gives the nonlinear current perpendicular to the THz electric field, the side-jump mechanism contributes to all photocurrents. For circular polarization, both BCD and side-jump mechanisms contribute to the nonlinear currents. [Note that for bulk tellurium the BCD contribution to the circular photocurrent along *C*_3_-axis was calculated in ref. (^37^).]

We have studied the direct current excited by polarized terahertz radiation in tellurene structures. The current, which is quadratic in the electric field, belongs to the class of nonlinear electron transport effects. Photocurrents sensitive to radiation helicity and orientation of the electric field vector as well as gate voltage were observed. We attribute the THz-induced currents to two mechanisms arising from the low spatial symmetry of 2D tellurene: the intrinsic Berry curvature dipole and extrinsic side jumps during momentum scattering. Importantly, the use of THz frequencies facilitates the observation of photocurrents, which are sensitive to the orientation of the ac electric field, as well as to the direction of its rotation.

## Supporting Information Available

Device fabrication and characterization, further experimental results, tables with fitting coefficients, the phenomenological description, and the skew scattering mechanism (PDF)
