# Supplementary material for: Terahertz Radiation Driven Nonlinear Transport Phenomena in Two-Dimensional Tellurene
Source: Nano Lett. 2024 Dec 25;25(1):476–82. doi: 10.1021/acs.nanolett.4c05279 (PMC11719634; doi:10.1021/acs.nanolett.4c05279)
Supplement: Supplementary file 1 — nl4c05279_si_001.pdf [file nl4c05279_si_001.pdf]

Supporting Information for Terahertz radiation driven nonlinear transport phenomena in two-dimensional tellurene

E. Mönch,[†] M. D. Moldavskaya,[†] L. E. Golub,[†] V. V. Bel'kov,[†] J. Wunderlich,[†,‡] D. Weiss,[†] J. V. Gumenjuk-Sichevska,[¶,§] Chang Niu,[‖,⊥] Peide D. Ye,[‖,⊥] and S. D. Ganichev[*,†,#]

†*Physics Department, University of Regensburg, 93040 Regensburg, Germany*
‡*Institute of Physics, Czech Academy of Sciences, Cukrovarnická 10, 162 00 Praha 6, Czech Republic.*
¶*Johannes Gutenberg-University Mainz, D-55128 Mainz, Germany*
§*V. Lashkaryov Institute of Semiconductor Physics, National Academy of Science, 03028, Kyiv, Ukraine*
‖*Elmore Family School of Electrical and Computer Engineering, Purdue University, West Lafayette, Indiana 47907, United States*
⊥*Birck Nanotechnology Center, Purdue University, West Lafayette, Indiana 47907, United States*
#*CENTERA Labs, Institute of High Pressure Physics, PAS, 01 - 142 Warsaw, Poland*

E-mail: sergey.ganichev@ur.de

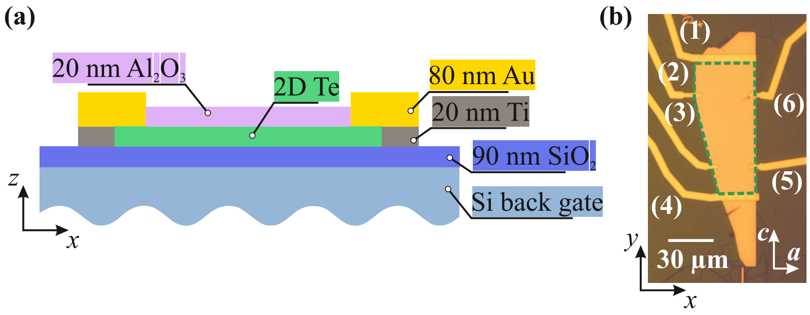

Figure S1: Panel (a) shows a typical cross section of the 2D Te devices under investigation. Panel (b) shows an optical microphotograph of sample #B and the corresponding contact numbering. The green dashed pentagon highlights the Hall bar.

Device fabrication

Our tellurene samples were made by the method introduced in Ref. (1). First, 0.5 g of polyvinylpyrrolidone (PVP) (Sigma-Aldrich) and 0.09 g of Na_2TeO_3 (Sigma-Aldrich) were dissolved in 32 ml double-distilled water. Then, 3.33 ml of aqueous ammonia solution (25-28%, weight by weight %) and 1.67 ml of hydrazine hydrate (80%, weight by weight %) were added under magnetic stirring to form a homogeneous solution. The mixture was sealed in a 50 ml Teflon lined stainless steel autoclave and heated at 180°C for 20 hours, then allowed to cool naturally to room temperature. The synthesized 2D Te flakes with a thickness of $\approx$ 20 nm were rinsed twice in water before being transferred to a 90 nm SiO_2/Si substrate, which served as a back gate, using the Langmuir-Blodgett method. Electron beam lithography was then used to fabricate six-terminal Hall-bar structures, and electron beam evaporation was used to deposit the metal contacts consisting of 20/80 nm Ti/Au. To cap the Te flakes, a 20 nm thick layer of Al_2O_3 was deposited on top by atomic layer deposition using $(CH_3)_3Al$ (TMA) and H_2O as precursors at 200°C. Figure S1 shows the cross-section [panel (a)] and a micrograph [panel (b)] of sample #B. Its parameters and layer thicknesses are very similar to that of sample #A.

Figure S2 shows exemplarily the characterization of the as-grown Te flake of sample #A, including SEM [panel (a)], TEM [panel (b)], and Raman [panel (c)]. These results are consistent with our previous work, see, e.g., Ref. (2). We observed a uniform surface within the whole flake and good crystallinity, indicating good quality of the sample. The high-resolution scanning transmission electron microscopy HR-STEM image and Raman spectrum also provide evidence of good crystallinity. Here, the sample is tellurium in a two-dimensional form, where the quantum Hall effect is observed in similar samples[1] indicating the two-dimensional nature of the carriers in the flake. Furthermore, the observed circular and linear photocurrents generated by normal incident radiation in unbiased samples unambiguously demonstrate the reduction of symmetry to the lowest one (C_1), which also supports that the response is obtained from 2D Te.

Additional data

Additional data include:

- Figure S3 illustrates a power dependence of the photocurrent J^y obtained in sample #A. It demonstrates a clear linear behavior upon the applied radiation power, P being proportional to the laser beam intensity I. The latter in turn is proportional to E_0^2, where E_0 is the electric field amplitude of the applied THz radiation.

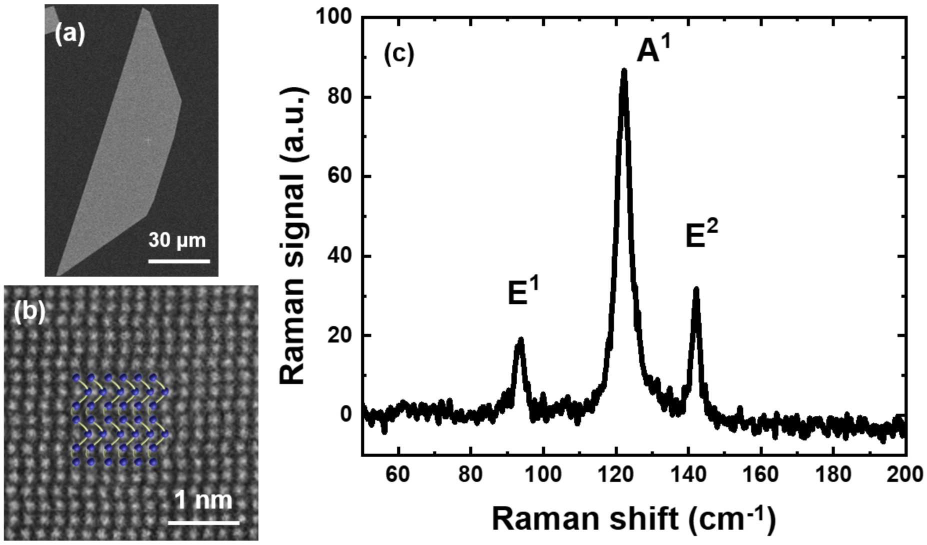

Figure S2: Material characterization of sample #A. (a) SEM image, (b) HR-STEM image showing the crystal structure, and (c) Raman spectrum.

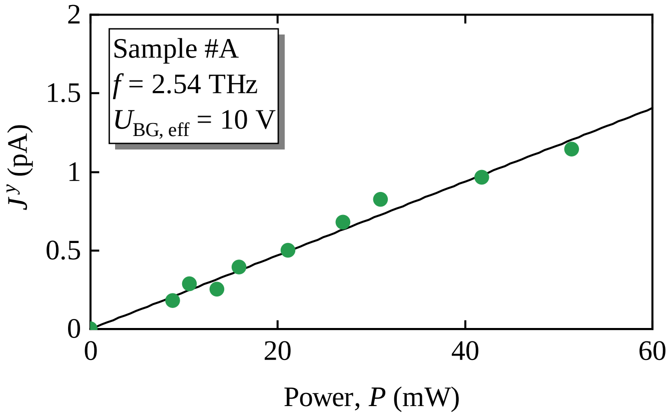

Figure S3: Power dependence of the photocurrent J^y measured along the Hall bar at room temperature. The electric field of the radiation was directed across the Hall bar. The solid line corresponds to a linear fit.

- Figure S4 shows the gate voltage dependence of the circular and polarization independent photocurrent projections measured in $x-$ and x- directions. The fit coefficients used for sample #A are listed in Tab. S1. For convenience the values are related to the figure number and the corresponding back gate voltage.

- Figure S5 shows the dependence of the photocurrent on the orientation of the radiation electric field vector in respect to the x-axis measured in x- and x- directions. The fit coefficients used for sample #A are listed in Tab. S1. For convenience the values are related to the figure number and the corresponding back gate voltage.

- Figures S6 - S8 present the dependencies of the photocurrents on the radiation helicity, the polarization orientation, and on the back gate voltage, respectively in both x- and y-directions. The fit coefficients used for sample #B are listed in Tab. S2. For convenience the values are related to the figure number and the corresponding back gate voltage.

Table S1: Coefficients of the fits after Eqs. (3) and (4) of the main text for sample #A. The first two columns give the corresponding figure numbers (of the main text and the SI) and the effective back gate voltages of the fitted curves.

Figure No.	$U_{\mathrm{BG,eff}}$ (V)	J_0 (pA/W)	J_{L1} (pA/W)	J_{L2} (pA/W)	J_{circ} (pA/W)
Figure 2(a)	-11	-7.7	24.3	2.0	-18.7
Figure 2(b)	-5	-8.6	6.7	2.0	-3.6
Figure 2(c)	16	51.4	-51.2	-19.1	12.3
Figure 2(d)	10	31.9	-19.7	5.3	-11.8
Figure 2(e)	10	17.1	-23.1	10.9	-35.4
Figure 4(a)	-11	-29.9	27.4	11.3	–
Figure 4(b)	-8	-21.1	8.8	6.3	–
Figure 4(c)	1	12.0	4.2	0.6	–
Figure 4(d)	4	28.8	7.6	-11.1	–
Figure 4(e)	10	57.4	9.8	-32.7	–
Figure 4(f)	16	57.2	4.9	-24.1	–
Figure S5(a)	1	-16.8	13.1	7.4	–
Figure S5(b)	1	12.0	4.2	0.6	–

Table S2: Coefficients of the fits after Eqs. (3) and (4) of the main text for sample #B. The first two columns give the corresponding figure numbers (of the main text and the SI) and the effective back gate voltages of the fitted curves.

Figure No.	$U_{\mathrm{BG,eff}}$ (V)	J_0 (pA/W)	J_{L1} (pA/W)	J_{L2} (pA/W)	J_{circ} (pA/W)
Figs. 2(f) and S6(a)	-2.0	8.6	-8.4	-20.6	-22.0
Figure S6(b)	-8.5	2.4×10^4	-1.7×10^4	-8.2×10^3	3.3×10^3
Figure S7(a)	0.5	-49.6	-50.0	-67.6	–
Figure S7(b)	-3.5	-2.6×10^2	4.7×10^2	-1.2×10^3	–
Figure S7(c)	-8.5	1.8×10^4	-1.1×10^4	-7.5×10^3	–

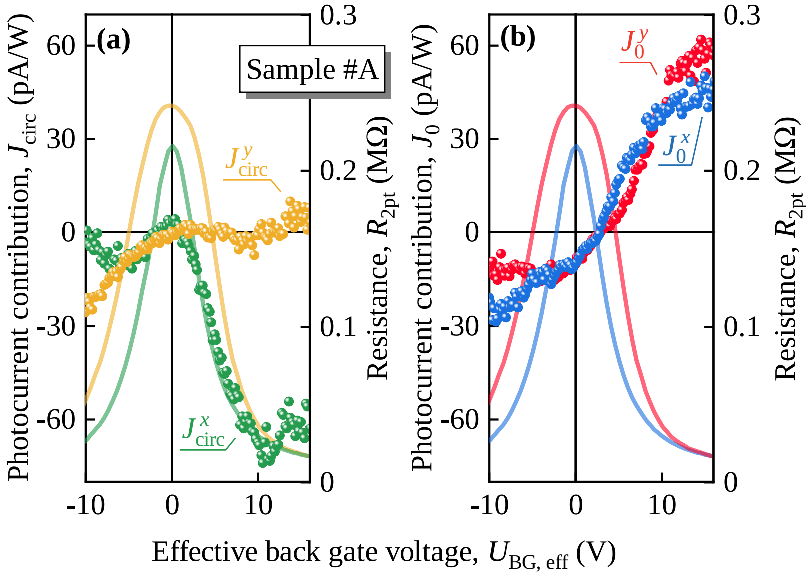

Figure S4: Back gate voltage dependencies of the circular ($J^{x,y}_{circ}$) and polarization independent ($J^{x,y}_0$) photocurrents measured across (J^x_{circ}, along the x-direction) and along (J^y_{circ}, along the y-direction) the Hall bar obtained at room temperature. The two-terminal resistances are shown by correspondingly colored curves (right axes).

Photocurrent polarization dependencies analyzed with Stokes parameters

Equation (3) and (4) in the main text describe the polarization dependence of the observed photocurrent. The polarization state of radiation is given by four Stokes parameters, which describe the degree of linear polarization in x, y coordinates (P_{L1}), in coordinate system rotated by 45° in respect to x, y (P_{L2}), the degree of circular polarization (P_{circ}), and the radiation intensity (P_0), see Ref. (3). To fit the experimental data, we used a linear combination of these parameters with different weights as variable parameters. Equations (3) and (4) are used to fit the polarization dependencies of the photocurrent obtained by rotation of $\lambda/4$ and $\lambda/2$ plates, respectively. As introduced in the section "Theory and discussion" of the main text, in the $\lambda/4$ setup the polarization degrees change after $P_{circ} = \sin(2\varphi)$, $P_{L1} = (\cos 4\varphi + 1)/2$, $P_{L2} = \sin(4\varphi)/2$, where φ is the angle of rotation of the $\lambda/4$ plate in respect to the radiation electric field vector of the THz laser, see Ref. (4). In the $\lambda/2$ plate setup, the Stokes parameters describing the orientation of the radiation electric field vector vary as $P_{L1} = \cos(2\alpha)$ and $P_{L2} = \sin(2\alpha)$, where α is the azimuth angle in respect to x-direction, see Fig. 1(c) in the main text.

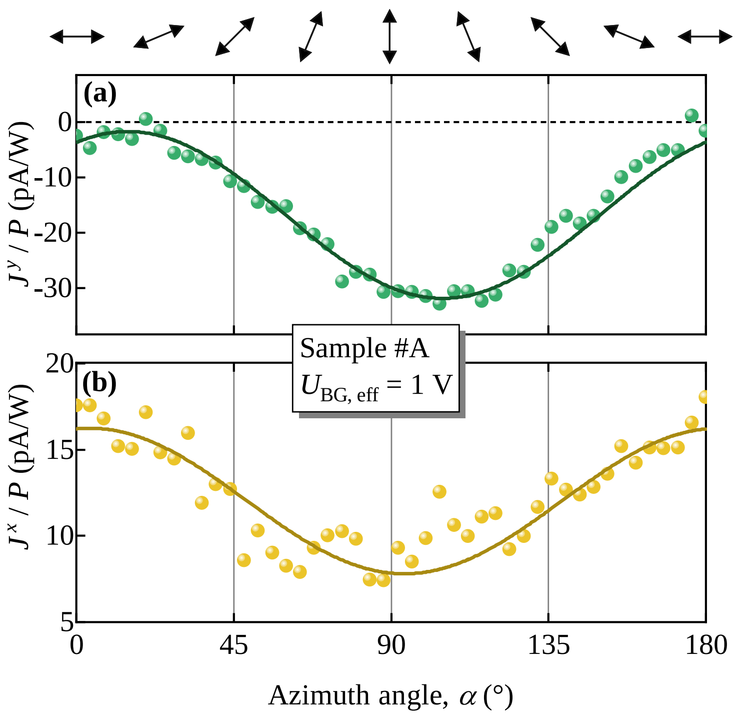

Figure S5: Photocurrents J^y, panel (a), and J^x, panel (b), as a function of the azimuth angle α defining the orientation of the electric field of linearly polarized radiation. The data are shown for $U_{\text{BG,eff}} = 1$ V. The circles show the measured traces, whereas the solid lines show the corresponding fits after Eq. (4) of the main text. The fit coefficients are given in Tab. S1. The symbols on top illustrate polarization states for several azimuth angles α.

Note that for right- ($\sigma^+, \varphi = 45°$) and left-handed ($\sigma^-, \varphi = 135°$) polarization the last two terms in Eq. (3) vanish and only circular and polarization independent photocurrents contribute to the signal.

Gate voltage dependence analysis

We now analyze the gate dependencies of the polarization-independent and helicity-driven nonlinear currents. We start with J_0 presented in Figs. 3(b) of the main text and S4(b). Far from the CNP, this current is formed in the valence band (high negative $U_{\text{BG,eff}}$) or in the conduction band (high positive $U_{\text{BG,eff}}$). The finding that J_0 has opposite signs for holes and electrons [Fig. S4(b)] is due to the fact that the current is odd in the carrier charge [$\propto q^3$, see Eq. (7) and text immediately after]. Consequently, the current must be zero for a given gate voltage. Tellurene is characterized by a strong electron-hole asymmetry. Thus, the photocurrent magnitudes in the valence and conduction bands are different because they are defined not only by the carrier density but also by the band parameters and scattering details. Consequently, the zero of the total current must not be at the CNP. The data in Figs. 3(b) and S4(b) reveal that at the CNP J_0 is dominated by the photocurrent generated in the valence band, and only at positive back gate voltage, which leads to a significant increase in electron density, is overcome by the contribution from the conduction band. We now discuss the gate dependence of J_{circ}. While Fig. 3(a) of the main text shows that it approaches zero near the CNP, the individual components shown in Fig. S4(a) do not change their sign. This at first sight surprising result is caused by the microscopic mechanism of J_{circ} formation. It crucially requires the structure inversion asymmetry (SIA), which in tellurene is defined by the internal and external SIA being proportional to the gate voltage. Consequently, the resulting current given by

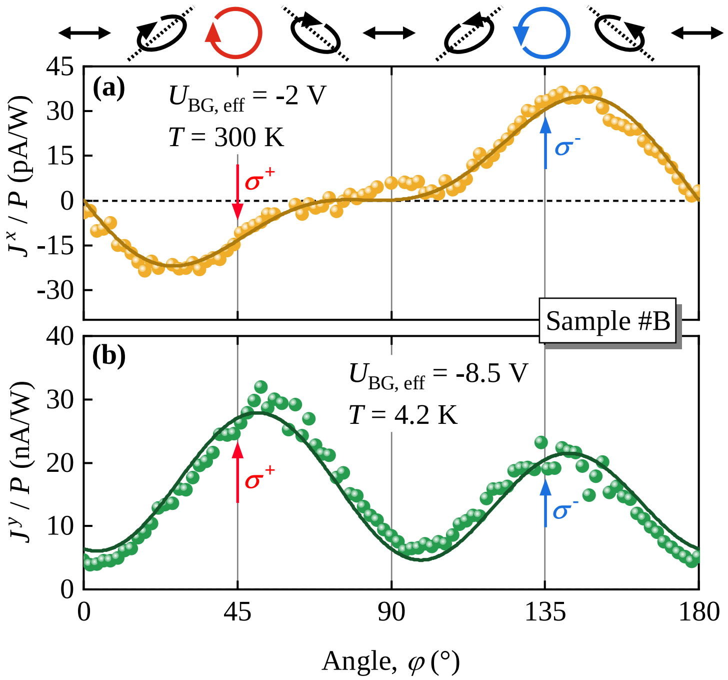

Figure S6: Photocurrents J^x, panel (a), and J^y, panel (b), measured in sample #B as a function of the angle φ defining the degree of circular polarization. The former was measured at $T = 300$ K, and the latter was obtained at $T = 4.2$ K. The circles represent the measured traces, whereas the solid lines show the corresponding fits after Eq. (3) of the main text. The fit coefficients are given in Tab. S2. The symbols on top illustrate polarization states at several angles φ, and the red and blue arrows label the right-handed (σ^+) and left-handed (σ^-) circular polarized radiation, respectively.

a product of q^3 and $U_{\text{BG,eff}}$, must not change its sign as a function of $U_{\text{BG,eff}}$. Apart from these macroscopic parameters, the formation of the photocurrent at indirect intraband optical transitions is determined by complex band spin splittings in tellurene, resulting in particular, in the camel-back structure of the valence band. Note that, the observed cancellation of the photocurrent in sample #A at the CNP in Fig. 3(a) is occasional. In fact, in contrast to sample #A, a significant J_{circ} is observed at the CNP in sample #B, see Fig. S8, which may be caused by a different built-in SIA in these samples.

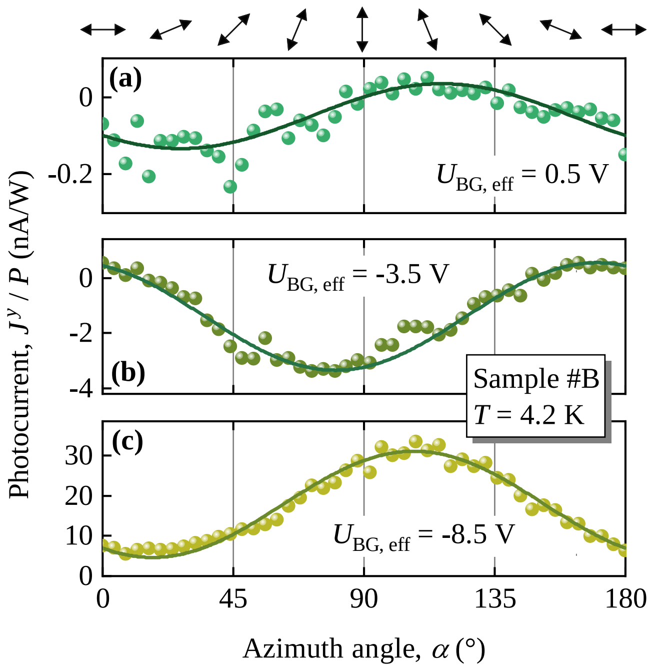

Figure S7: Azimuth angle dependencies of the photocurrent J^y (colored circles) obtained for several examples of effective back gate voltages. Selected polarization states are shown on top of the figure. The solid lines represent the corresponding fits after Eq. (4) of the main text. The fit coefficients are given in Tab. S2.

Phenomenological description

In this section we address the relation between both sides of Eq. (2) in the main text. In the first line of Eq. (2), $E_\nu^* = E_\nu^*(\omega) = E_\nu(-\omega)$ is the complex conjugate of E_ν, and the expansion coefficients $\sigma^{(2)}_{\alpha\mu\nu}$ form a third rank tensor. It is convenient to write the external product $E_\mu E_\nu^*$ in the first line of Eq. (2) as the sum of a symmetric and an antisymmetric product

$$E_\mu E_\nu^* = (E_\mu E_\nu^* + E_\mu^* E_\nu)/2 + (E_\mu E_\nu^* - E_\mu^* E_\nu)/2\,. \tag{S1}$$

This decomposition of $E_\mu E_\nu^*$ is equivalent to a splitting into real and imaginary parts. The symmetric term is real while the antisymmetric term is purely imaginary. Due to contraction of the tensor $\sigma^{(2)}_{\alpha\mu\nu}$ with $E_\mu E_\nu^*$ the same algebraic symmetries are projected onto the last two indices of $\sigma^{(2)}_{\alpha\mu\nu}$. The real part of $\sigma^{(2)}_{\alpha\mu\nu}$ which is denoted as $\chi_{\alpha\mu\nu}$ is symmetric in indices μ and ν ($\chi_{\alpha\mu\nu} = \chi_{\alpha\nu\mu}$), whereas the imaginary part is antisymmetric. Antisymmetric tensor index pairs can be reduced to a single pseudovector index using the Levi-Civita totally antisymmetric tensor $\delta_{\rho\mu\nu}$. Applying this simplification, we obtain for the current due to the antisymmetric part of

$$\sigma^{(2)}_{\alpha\mu\nu}(E_\mu E_\nu^* - E_\mu^* E_\nu) = \mathrm{i}\gamma_{\alpha\rho}\delta_{\rho\mu\nu}E_\mu E_\nu^* = \gamma_{\alpha\rho}\mathrm{i}[\boldsymbol{E}\times\boldsymbol{E}^*]_\rho\ , \tag{S2}$$

with the real second rank pseudotensor $\gamma_{\alpha\rho}$, and $[\boldsymbol{E}\times\boldsymbol{E}^*]$ stands for the vector product of the vectors $\boldsymbol{E}$ and $\boldsymbol{E}^*$ which is nonzero for elliptical polarization. In summary, for the total photocurrent we find

$$j_\alpha = \chi_{\alpha\mu\nu}(E_\mu E_\nu^* + E_\mu^* E_\nu) + \gamma_{\alpha\rho}\mathrm{i}[\boldsymbol{E}\times\boldsymbol{E}^*]_\rho\ , \tag{S3}$$

which is the second line of Eq. (2). Note that, in our experimental setup, the μ and ν axes are in the $(x,\,y)$ plane and the direction of ρ is perpendicular to the plane.

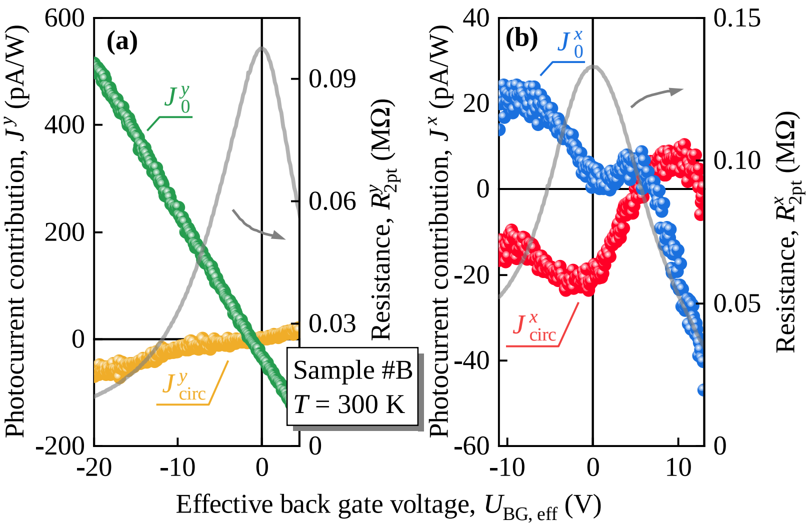

Figure S8: Back gate voltage dependencies of the circular and the polarization independent photocurrent contributions along the y, panel (a), and the x, panel (b), directions. The two-terminal resistances are shown by the gray shaded curves (right axes).

Skew scattering mechanism

The skew-scattering is caused by the fact that, in noncentrosymmetric systems, the scattering probability does not satisfy the principle of detailed balance: the probability of $\boldsymbol{k}' \to \boldsymbol{k}$ process, $W_{\boldsymbol{kk}'}$ differs from that of inverse process, $W_{\boldsymbol{k}'\boldsymbol{k}}$. Therefore it is possible to make a splitting to the symmetric and asymmetric parts: $W_{\boldsymbol{kk}'} = W^0_{\boldsymbol{kk}'} + W^{\rm sk}_{\boldsymbol{kk}'}$, where $W^{0,\rm sk}_{\boldsymbol{kk}'} = \pm W^{0,\rm sk}_{\boldsymbol{k}'\boldsymbol{k}}$.[5] The skew-scattering induced correction to the distribution function is obtained by two iterations of the kinetic equation in powers of the electric field $\boldsymbol{E}_\omega$ and one iteration in $W^{\rm sk}_{\boldsymbol{kk}'}$. Then the skew-scattering contribution to the photocurrent is calculated by Eq. (7) of the main text with an ordinary velocity. In systems of C_1-symmetry it gives rise to all contributions in Eq. (5) of the main text.

At high frequency used in the experiments we have $\omega\tau > 1$, where τ is a transport relaxation time. Therefore, the skew-scattering based mechanism of the circular photocurrent is suppressed and yield negligible contribution in respect to the BCD and side-jump driven photocurrents. Indeed, this is valid for both terms in the skew scattering probability. A so-called conventional skew scattering occurs with non-Gaussian disorder and appears in the third order in the scattering potential. It is present at low temperatures for scattering by impurities, but is absent for acoustic phonon scattering when single-phonon processes are dominant.[6,7] It is also valid, for another, the so-called intrinsic skew scattering, occurs at any potential and is proportional to the fourth power of the disorder potential. Summarizing, at high frequencies, $\omega\tau \gg 1$, the intrinsic skew scattering contribution to circular photocurrent is suppressed as it scales with $(\omega\tau)^{-2}$ of the BCD and side-jump contribution.[8]

References

(1) Qiu, G.; Niu, C.; Wang, Y.; Si, M.; Zhang, Z.; Wu, W.; Ye, P. D. Quantum Hall effect of Weyl fermions in n-type semiconducting tellurene. *Nat. Nanotechnology* **2020**, *15*, 585–591.

(2) Niu, C.; Qiu, G.; Wang, Y.; Tan, P.; Wang, M.; Jian, J.; Wang, H.; Wu, W.; Ye, P. D. Tunable Chirality-Dependent Nonlinear Electrical Responses in 2D Tellurium. *Nano Lett.* **2023**, *23*, 8445–8453.

(3) Saleh, B. E. A.; Teich, M. C. *Fundamentals of Photonics*; John Wiley and Sons Ltd., 2019.

(4) Bel'kov, V. V.; Ganichev, S. D.; Ivchenko, E. L.; Tarasenko, S. A.; Weber, W.; Giglberger, S.; Olteanu, M.; Tranitz, H. P.; Danilov, S. N.; Schneider, P.; Wegscheider, W.; Weiss, D.; Prettl, W. Magneto-gyrotropic Photogalvanic Effects in Semiconductor Quantum Wells. *J. Phys. Condens. Matter* **2005**, *17*, 3405.

(5) Otteneder, M.; Hubmann, S.; Lu, X.; Kozlov, D. A.; Golub, L. E.; Watanabe, K.; Taniguchi, T.; Efetov, D. K.; Ganichev, S. D. Terahertz Photogalvanics in Twisted Bilayer Graphene Close to the Second Magic Angle. *Nano Lett.* **2020**, *20*, 7152–7158.

(6) Glazov, M. M.; Golub, L. E. Skew Scattering and Side Jump Drive Exciton Valley Hall Effect in Two-Dimensional Crystals. *Phys. Rev. Lett.* **2020**, *125*, 157403.

(7) Glazov, M. M.; Golub, L. E. Valley Hall effect caused by the phonon and photon drag. *Phys. Rev. B* **2020**, *102*, 155302.

(8) Golub, L. E.; Ivchenko, E.; Spivak, B. Semiclassical theory of the circular photogalvanic effect in gyrotropic systems. *Phys. Rev. B* **2020**, *102*, 085202.
